# Noble Metal Based Alloy Nanoframes: Syntheses and Applications in Fuel Cells

**DOI:** 10.3389/fchem.2019.00456

**Published:** 2019-07-03

**Authors:** Farhat Nosheen, Tauseef Anwar, Ayesha Siddique, Naveed Hussain

**Affiliations:** ^1^Department of Chemistry, University of Education, Jauharabad, Pakistan; ^2^Department of Physics, The University of Lahore, Lahore, Pakistan; ^3^Sulaiman bin Abdullah Aba Al-Khail-Centre for Interdisciplinary Research in Basic Sciences, International Islamic University Islamabad, Islamabad, Pakistan; ^4^State Key Laboratory of New Ceramics and Fine Processing, School of Materials Science and Engineering, Tsinghua University, Beijing, China

**Keywords:** nanoframes, fuel cells, alloy, nanocages, multimetallic

## Abstract

Noble metal nanostructures are being used broadly as catalysts for energy conversion in fuel cells. To overcome the future energy crises, fuel cells are anticipated as clean energy sources because they can be operated at low temperature, their energy conversion is high and their carbon release is almost zero. However, an active and stable electrocatalyst is essential for the electrochemical reactions in fuel cells. Therefore, properties of the nanostructures greatly depend on the shape of the nanostructures. Individual as well as interaction properties are greatly affected by changes in the surface area of the nanostructures. By shape controlled synthesis, properties of the nanostructures could be further enhanced by increasing the surface area or active sites for electrocatalysts. Therefore, an efficient approach is needed for the fabrication of nanostructures to increase their efficiency, activity, or durability in fuel cells by reducing the usage of noble metals. Different types of hollow nanostructures until now have been prepared including nanoboxes, nanocages, nanoshells, nanoframes (NFs), etc. NFs are the hollow unique three-dimensional structure which have no walls—they only contain corners or edges so they have large surface area. In electrocatalytic reactions, the molecules involved in the reaction can easily reach the inner surface of the nanoframes, thus noble metals' utilization efficiency increases. NFs usually have high surface area, greater morphological and compositional stabilities, allowing them to withstand harsh environmental conditions. By considering the current challenges in fabrication of noble metal based alloy NFs as electrocatalysts, this review paper will highlight recent progress, design, and fabrication of noble metal alloy NFs through different strategies—mainly photocatalytic template, electrodeposition, Kirkendall effect, galvanic replacement, chemical/oxidative etching, combination of both and other methods. Then, electrochemical applications of NFs in fuel cells toward formic acid, methanol, ethanol, oxygen reduction reaction as well as bifunctional catalyst will also be highlighted. Finally, we will summarize different challenges in the fabrication of highly proficient nanocatalysts for the fuel cells with low cost, high efficiency and high durability, which are the major issues for the highly commercial use of fuel cells in the future.

## Introduction

Noble metal nanostructures are being used broadly as catalysts for energy conversion in fuel cells. To overcome the future energy crises, fuel cells are anticipated as clean energy sources. Noble metals such as platinum (Pt) as well as palladium (Pd) are the most important and effective catalysts in fuel cells, but increasing the catalyst efficiency as well as cost reduction of catalysts is a great challenge. By tuning the size, composition, and morphology, electrocatalytic activity could be enhanced (Ding et al., 2012; Wang et al., 2013, 2015a; Zhang et al., 2018b). Recent research efforts have been focused on (1) the addition of cheap transition metals to decrease the cost and improve the electronic effect of Pt, (2) tuning the morphologies with high index facets, ultrathin walls and 3D surface accessibility, (3) NF architecture which shows the 3D surface with high catalytic surface area as well as large utilization of precious metals, (4) composition segregation of Pt on the edges and corners of NFs to increase the Pt usage. Furthermore, it is very necessary to fabricate the novel nanocatalyst with both higher activity as well as stability.

Pt content could be reduced without compromising the catalytic properties by changing the solid nanostructures into hollow nanostructures with ultrathin walls and with controlled surface composition. The advantage of hollow structures over the solid particles is that Pt atoms in solid nanoparticles are unexploited because they cannot participate in electrocatalytic reactions. However, the empty interior of nanocages and nanoframe allows the reactants to interact with 3D interior and exterior surfaces in catalytic reactions (Fang et al., 2015). Moreover, atomic scale ultrathin walls below 2 nm can further improve the electrocatlytic applications. In previous studies, a lot of hollow as well as nanocage structures have been fabricated, but NF architectures—especially bi-and multimetallic NFs—have not received more attention. Attaining NFs with controlled size, highly open structure, 3D surface area, control thickness, and stable shell is a more difficult task. In recent years, some groups have been interested in the atomic scale synthesis of bi- and multimetallic NFs due to their excellent electrocatalytic activity as well as durability in fuel cells. In this review, we will highlight the state-of-the-art accomplishments on NFs and will discuss the growth mechanism, which includes the photocatalytic template method, electrodeposition, Kirkendall effect, galvanic replacement reaction, oxidative/chemical etching, and others. In previous years, researchers have fabricated the single component noble metal hollow, nanocages and NFs, but few have focused on the fabrication of noble metal based bimetallic and multimetallic NFs for fuel cells.

## Synthetic Methodologies for Nanoframes

### Photocatalytic Template Synthesis

Template based synthesis has been widely employed for the synthesis of various hollow nanostructures (Bai et al., 2011). Photocatalytic-based hard and soft template synthesis has been used by a few groups to synthesize Pt nanocages. For example, Shi et al. developed Pt nanocages by using the liposomes as a template in which photocatalyst molecules were present. In this protocol, firstly hydrophobic Sn^IV^ octaethylporphyrin (SnOEP) has been integrated into unilamellar liposomes. After that, platinum seeds were formed due to photo-catalytic reduction of Pt salts and converted in the form of dendritic Pt-nanosheets. Then these dendritic nanosheets combined with each other inside the bilayer and evolved into nanocages. The dendritic nanosheets after combining with each other adopted the spherical structure of the liposomal template with a 2 nm thick wall and 200 nm nanocages. The synthesized Pt nanocages contain hollow voids and additional porosity, which may be beneficial for extensive applications (Shi et al., 2015). Moreover, Wang et al. developed 3D Pt nanocages by simple photocatalytic template procedure. The synthetic procedure was carried out in the presence of TiO_2_ NPs that not only worked as photocatalyst but also as template. In synthesis, Pt uniform interlinked branches first formed by the increased amount of Pt precursors and reducing the intensity of light. Then, these nanobranches were photo-deposited on a TiO_2_ template by irradiating the UV light without any surfactants or capping agents. After template removal, 3D Pt nanocages were attained with high surface area as well as more active sites showing them to be an active catalyst for MOR. By the photocatalytic template method, nanocages of different shapes and sizes could be synthesized by varying the exposure of light, amount of metal precursors, and size of template (Zhu et al., 2015).

### Electrodeposition Method

The electrodeposition method consists of the deposition of a substance on substrate by introducing an electric current. This method is used for the fabrication of solid particles with very low porosity. However, for the creation of metal based NFs, this technique is also coupled with other strategies like chemical etching as well as electroleaching. For instance, Moghimi et al. developed FeNi NFs by the electrodeposition and electroleaching technique (Moghimi et al., 2013). The electrodeposition technique combined with chemical etching could be employed to fabricate various NFs. For example, Torimoto et al. synthesized Au NFs by the preferred electrodeposition of Au on the edges and corners of Ag cubic particles treated with the 1-octanethiol, and then chemical etchants (hydrogen peroxide and sulfuric acid) were used to chemically etch Ag particles. In this method, Ag has been used as a template, and dissolution of Ag template has not been observed during the deposition process of Au. This strategy could be used for the fabrication of NFs where simultaneous dissolution of the template needs to be avoided during electro-deposition of second material. Moreover, electrodeposition favors the formation of metal NPs irrespective of metal and their reduction potential. This method has rarely been used for synthesis of noble metal-based alloy nanoframes (Okazaki et al., 2009).

### Kirkendall Effect

**Figure 1:**
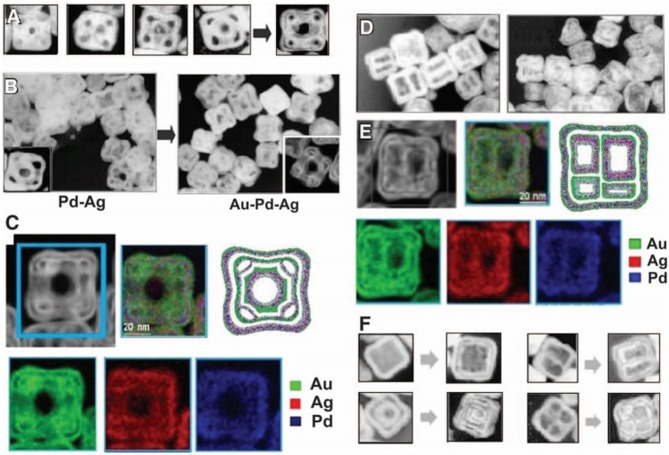
**(A)** TEM images illustrating the stages of formation of hollow nanostructures by sequential action of galvanic replacement and Kirkendall effect. **(B)** TEM images showing the nanostructures dominated by galvanic replacement using palladium (left) and by Kirkendall effect using gold (right). **(C)** HAADF-STEM image and corresponding EDX elemental maps. **(D)** TEM images of multichambered NPs. **(E)** HAADF-STEM detail and corresponding EDX elemental maps. **(F)** TEM images of different structures produced by sequential action of galvanic replacement and Kirkendall effect. Adapted with permission from González et al. (2011) Copyright 2011, American Association for the Advancement of Science.

The Kirkendall effect is a vacancy mediated technique which is based on the net mass flow of faster diffusing species, and it is balanced by the opposing flow of vacancies. This method is useful for the synthesis of hollow and porous NCs because it is a diffusion procedure. González et al. used sequential galvanic replacement reaction or the Kirkendall effect at room temperature to synthesize polymetallic hollow nanocrystals (NCs) with complicated shapes and compositions such as trimetallic Pd-Au-Ag nanoboxes (González et al., 2011), as shown in Figure 1. Han et al. developed the Cu_3_Pt NFs with unique morphology by a transformation process. The Kirkendall effect is not commonly used for noble metal alloy NFs, so we will focus more on other methods.

### Galvanic Replacement Reaction

The galvanic replacement reaction is extensively employed to synthesize metal NCs with hollow interiors such as nanocages, nanoboxes, and NFs. The template oxidizes and dissolves during the galvanic replacement reaction, meanwhile metal ions reduce, and deposit on the template surface. The discrepancy of the two metals in redox potential is the driving force of the galvanic replacement. However, the structure of the NCs may be controlled by tuning the metal to salt ratio, quantity and the shape of the template. The same sacrificial template facets have the simultaneous removal of template and the deposition of atoms on all the facets. In the template containing distinct facets, the galvanic replacement reaction adopted the facet selectively. Atoms are dissolved at higher surface energy and deposited on the lower energy facets. In addition, template based, and one pot strategy are the two main methods to carry the galvanic replacement reaction (Xia et al., 2013).

### Template Based Galvanic Replacement Method

Ag metal has a lower reduction potential in comparison with the other metals such as Pd, Au, etc. Due to this property, it is easy to remove the atoms by the galvanic replacement reaction, and it makes Ag the most efficient sacrificial template for the synthesis of hollow nanostructures. For instance, Mahmoud and El-Sayed have developed the double shell nanocages of different sizes with controlled thickness and used the Ag as template (Mahmoud and El-Sayed, 2012). In this protocol, the Ag template has been employed because at elevated temperatures single-crystalline Ag particles formed, which may produce the single crystalline hollow structures in cases where the lattice of Ag and other metals match well. The size of templates was controllable from a few to 250 nm, which produced the hollow structures of different sizes. Synthesis of Ag template could be done with high yield. PVP is a good capping agent for Ag NC synthesis and it is appropriate for other metals as well, therefore the capping of the Ag template is also suitable for the synthesis of hollow structures and prevents their aggregation. In addition, the bowl-like morphology of PtAuAg nanocages was synthesized by using the seed mediated and galvanic replacement procedure (Xu et al., 2018) in which Ag seeds were used as a template. However, experiments in different optimized conditions were able to fabricate the structures which have time dependence and also depend on cetyltrimethylammonium chloride (CTAC). Due to their structure and synergetic effect of Pt, Au, and Ag, the final PtAuAg nanocatalyst exhibits higher catalytic performance. Furthermore, similar to the Ag template, Pd NCs were also used as sacrificial templates for the fabrication of PdPt hollow nanocages via the galvanic reaction as well as the co-reduction method. In another report, Xia et al. developed PdPt nanocages through a co-reduction approach along hollow centers by a combination of galvanic replacement by using Pd nanocubes as a sacrificial template and K_2_PtCl_4_ in presence of citric acid. Meanwhile higher concentrations of bromide and ${\text{PtCl}}_{4}^{-2}$ at elevated temperatures accelerated the galvanic replacement and produced PdPt alloy nanocages (Zhang et al., 2011). However, by replacing the citric acid with the ascorbic acid (AA), PdPt nanodendrites were gained because of the faster depletion of Pt precursors and the decreased rate of the galvanic replacement reaction. For instance, Han et al. have fabricated PdPt octahedral and cubic nanocages with hollow interiors containing porous walls by using Pd octahedra and cubic NCs as sacrificial templates. Change in the ratio of AA could control the galvanic replacement reaction and gives the PdPd alloys with distinct shapes (Hong et al., 2012a).

In addition to metal templates, metal oxide NCs have also been used as a template. Chen et al. fabricated flower shaped PdCu nanocages by utilizing the corner etched Cu_2_O octaherda as a template through a facile and simple procedure. By adding the H_2_PdCl_4_, Cu_2_O were hybridized to generate the Cu_2_O@PdCu core shell, and etching of Cu_2_O was done by NH_3_.H_2_O. These flower shaped nanocages of PdCu were used for the MOR that shows the higher catalytic activity, excellent stability, and more poison tolerance. These alloy nanoarchitectures were developed by the galvanic reaction and disproportionation reaction (Chen et al., 2018). Similar work is also reported by the other research group, where they used Cu_2_O as a sacrificial template and distinct reducing agents to gain the cubic PtCu nanocage by redox reactions (Tian et al., 2014).

### One-Pot Galvanic Replacement Method

Using the pre-synthesized templates like Pd or Ag in the synthesis of hollow nanostructures has more limitations as the synthetic procedure requires more steps such as template synthesis, shell growth as well as template removal, which limits the practical use. Use of the noble metals like Pd or Ag as sacrificial template also wastes the expensive materials and the final morphology is limited to the initial shape of the parent templates. Alternatively, to synthesize metal alloy NFs through one pot procedure is more difficult as compared to monometallic NCs because the insertion of the second metal makes the reduction more difficult. The standard reduction potential of noble metals is higher in comparison with the non-noble metals, while for the alloy NC synthesis the reduction potential difference among the two metals should be decreased. For this purpose, some ligands could be used in order to decrease the reduction rate of the two metals. Another way to reduce the reduction potential is to combine the metal cations with coordinating agents like halide ions, which more strongly coordinate with the noble metals. In one report, Huang et al. (2009) introduced iodide ions to the mixture of Pd(acac)_2_]/[Pt(acac)_2_] in solution of DMF, which produced [PdI_4_]^2−^ as the most dominating precursors, and Pd reduced the Pt ions early. Furthermore, cubic PtCu_3_ nanocages were also developed by optimizing the reduction rate by the addition of cetyltrimethylammonium bromide (CTAB) as well as Pt and Cu species. From the above explanations, it is noticed that the CTAB in oleylamine works as a co-reductant, which tuned the Pt and Cu species reduction speed, and Cu ions are reduced before the Pt ions while the reduction rate of the Cu pair is more negative than the Pt pair (Xia et al., 2012).

**Figure 2:**
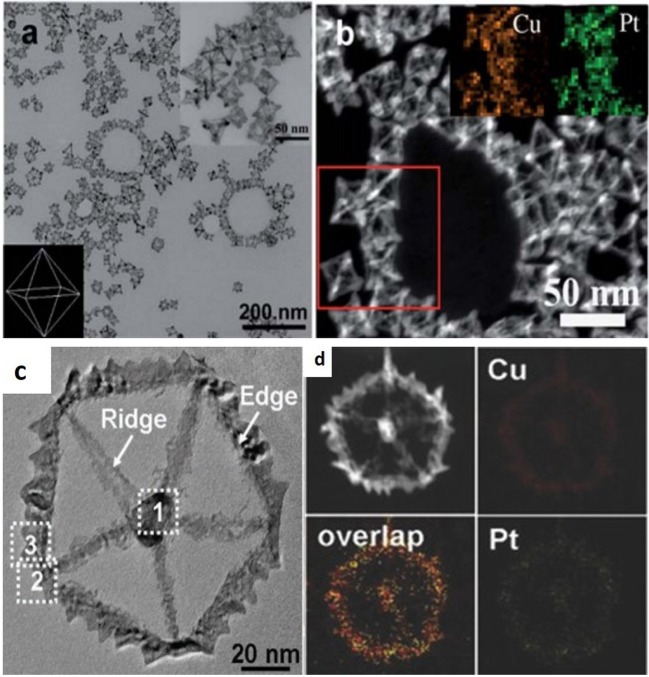
**(a)** TEM image of as-prepared octahedral Pt-Cu nanoframes the top-right inset shows a high-magnification TEM image of octahedral Pt-Cu nanoframes, and the bottom-left inset shows the corresponding model image. **(b)** HAADF-STEM image of octahedral Pt–Cu nanoframes; the inset shows the selected-area (red rectangle) element analysis maps of Pt (green) and Cu (orange). Modified with permission from Nosheen et al. (2013) Copyright 2013, Royal Society of Chemistry. **(c)** TEM images of PtCu nanoframes (NFs). **(d)** HAADF-STEM image and the corresponding EDS elemental mapping of a typical PtCu NF. Modified with permission from Zhang et al. (2016) Copyright 2016, WILEY-VCH Verlag GmbH & Co. KGaA, Weinheim.

Similarly, Zhang et al. reported the synthesis of PtCu yolk-cage like NCs through glycine mediated reduction kinetics. By changing the amount of glycine, morphology as well as composition of the NCs were controlled, and in this method glycine is used as a co-reductant and surface controller. Glycine may coordinate with the metal ions and when the medium is aqueous it strongly coordinates with Pt^4+^ instead of Cu^2+^. Subsequently, platinum ions' reaction to reduced copper nanocrysrtals by galvanic replacement leads to a Pt–Cu yolk cage structure. As compared to hollow structures or nanocages, NFs are open, have 3D surface accessibility and more active sites (Zhang et al., 2013). In addition to the above examples of nanocages, we also fabricated the single crystalline octahedral PtCu NFs, which involve the synergetic effect of OH and amine groups of ethanolamine and concentration of glycine also effect the reduction rate. However, here it was also observed that the coordinating ligands changed the reduction rate of metals and make galvanic replacement reaction more feasible (Nosheen et al., 2013) (Figures 2a,b). The same method was also adopted by Zhang's group, and they vary the time of reaction as well as temperature to gain the multiple twinned NFs. It is very rare to fabricate the multiple twined noble metal NFs (Figures 2c,d). Twin defects in the metal nanoarchitecture would modulate their electronic structure as well as surface reactivity and could enhance the catalytic performance. In case of twin configuration, the decahedron is the most compact pentagonal cyclic twined shape along the D_5_h symmetry and shows excellent catalytic applications. It is also observed that the twin defects act as a nucleation site which induces the generation of a highly anisotropic design and, as a result of symmetry breaking of the crystal lattice, gives more active sites for electrocatalysis. In addition, unique 5-fold twin PtCu NFs were also fabricated along nanothorn protruding from their edges. Meanwhile time dependence experimental analysis shows that pure Cu nanoctahedra were first generated. For the fabrication of anisotropic 5-fold twin PtCu NFs, galvanic replacement reaction among the Cu nanodecahedra as well as Pt precursors in solution and site-specific co-deposition of Pt as well as Cu atoms played a key role. Interestingly, these 5-fold twin PtCu NFs showed the excellent catalytic activity in terms of ORR as well as MOR in alkaline systems (Zhang et al., 2016).

Similarly, Huang et al. (2019) have developed the 3D RD Pt_26_Cu_74_ bound with multiple index facets through a facile solvothermal strategy. In this synthetic procedure, L-proline in combination with CTAC acted as a co-structure directing agent and modulated the nucleation as well as crystal growth rate. In comparison with the Pt_18_Cu_82_, Pt_50_Cu_50_, and commercial Pt/C catalyst, these Pt_26_Cu_74_ NFs showed a more enhanced catalytic performance toward ORR and HER because of their highly open nanostructure and synergetic effect of two metals. Polyhedral NFs like catalyst shows a more active and stable performance as compared to other NFs structures. For instance, Ding et al. fabricated the rhombic dodecahedral RD PtCu NFS along a highly open nanostructure in higher yield by the facile one pot method. In the synthetic procedure, a higher concentration of cupric chloride, and the combined use of glucose and oleylamine/oleic acid are responsible for the fabrication of PtCu NFs. TEM analysis proved that the formation procedure depends on initial formation of Cu nanoarchitectures and galvanic replacement of Cu nanoarchitecture with the Pt. Because of their highly open structure, these PtCu NFs showed excellent catalytic performance for MOR in comparison with the Pt/C as well as Pt catalysts (Ding et al., 2015).

### Chemical/Oxidative Etching

Etching can be defined as the selective removal of definite facets of NCs as well as less stable metals through alloys with modification of remaining atoms in order to control the shape, size, and composition. The porosity of NCs could be controlled by etchant strength and other conditions of reaction. However, most important advantage of etching process is to regulate the selectivity and rate of etching. Other parameters like the power of etchant, which consists of the chemical potential, concentration, and reaction temperature also play an important role, but selective etching includes a difficult mechanism, and it is more challenging to construct the open structure with a 3D surface by selective etching. Chemical etching has some disadvantages like surface atoms that may be removed on haphazard positions and an etching procedure that is sometimes very strong and is difficult to stop, meaning the structure could be damaged. In previous years, a lot of etchants have been used to fabricate nanoframe and nanocage structures (Xiong et al., 2005; Au et al., 2008; Zeng et al., 2009; Zhang et al., 2010; Wu et al., 2012; Xie et al., 2012). Moreover, it is a big challenge to control the etching process, and great efforts are still required to discover the path to choose a suitable etchant for the corrosion process. In addition, NFs gained by the etching method depend on precise anisotropic elemental distribution. However, compositional anisotropy of different components on the corners, edges, or facets is linked with geometrical elemental segregation (Liu et al., 2015).

### Two Step Etching

The formation of Pt and Pd based NFs is accomplished by the template-mediated growth of Pt atoms followed by the removal of template with acid etching or thermal annealing. Shape can be controlled by tuning the etching as well as regrowth rate. For instance, Wang et al. (2017b) fabricated palladium NFs by excavating the solid palladium NCs. In this synthetic procedure, etching, and regrowth steps are controlled. Different regrowth rates at corners/edges/faces and etching of Pd atoms at specific sites can be controlled by tuning the rate of etching and regrowth. Without reductant, etching rules over the process, which results in conversion of NCs with more defined structures like octahedral to cuboctahedral NCs. However, when a small quantity of reductant (HCHO) is used, the regrowth rate at corners and edge sites could be controlled, which is equivalent to the etching rate, but the regrowth rate at the face site is lower compared to the etching. So, palladium NFs can be gained if etching occurs on the faces only. By following this synthetic procedure, solid Pd cubic, octahedral, cuboctahedral, and concave cubic structures have been carved to analogous NFs. These excavated Pd NCs with a frame-like structure display excellent activity as well as durability in terms of formic acid oxidation reaction. Park and co-workers fabricated cubic Pt NFs and utilized Pd NPs as a template. Site selective coverage of bromide ions induced the selective deposition of Pt on edges/corners and Pt cubic NFs can be gained by discriminating etching of Pd cubes by using acid and H_2_O_2_ (Park et al., 2016).

### Nanoframes With Twinned Boundaries, High Index Facets, and Ultrathin Walls

The twin defects, ultrathin shell, and composition control could increase electrocatalytic properties. Zhang et al. synthesized nanocages by depositing a small number of atom layers of Pt as shell on Pd with more regular facets and etching the Pd template. On Pd NPs, Pt atoms were grown which are tuned from 1 to 6 atom layers by the maintained reaction conditions which effect the growth, diffusion, or dissolution behavior of Pt and Pd atoms (Zhang et al., 2015a). However, in the case of many structure sensitive reactions, introducing defects like twin boundaries may reduce the activation energy and enhance the catalytic performance. Therefore, concern has been focused toward the icosahedron, which has a higher density of twins in one NC. One icosahedron has 20 tetrahedrons in it and an individual tetrahedron is linked to another along the twin boundary, which endows the icosahedral nanocage as an efficient catalyst. He et al. (2016) have deposited Pt layer that is a few layers thick on the Pd to produce the icosahedral core shell NPs, and then by discriminating etching of Pd, icosahedral Pt enriches nanocages with layers that are a few atoms thick and defects were gained. In 2016, (Wu et al., 2014) synthesized platinum-based icosahedral nanocages which had a surface bounded by [111] facets as well as twin defects/boundaries and ultrathin walls of up to six atom layers. However, nanocages were derived from Pd@Pt_4.5L_ icosahedra by selective etching of the Pd core by using FeCl_3_ as well as KBr etchants. In the etching process, the multiple twinned structure was reserved and the Pt atom in the outer layer ws restructured to keep the original twinned structure with the ultrathin wall (Wang et al., 2016).

### Nanoframes With Compositional Segregation

Nanoscale compositional segregation, also called compositional anisotropy, has received more attention for the design of nanocatalysts because this procedure leads to 3D NCs with larger surface area as well as precise size and facet control. Depending on this procedure, the Pt component could be fully accessed by reactants and excellent electrocatalytic properties are obtained (Stamenkovic et al., 2006; Li et al., 2018).

In comparison to homogenous alloys, compositional segregation offers a stronger electronic effect of distinct metals, and many Pt sites are available. According to the structural as well as chemical perspective, the main benefit of the compositional segregated Pt NCs is that it allows the formation of highly open nanoarchitectures, nanocages, or NFs, which are the most promising class of nanostructure, with a 3D surface area as well as interconnected edges with high surface area and improved applications in fuel cells. Now researchers are focusing on the synthetic procedure for gaining compositional segregated Pt based nanocatalysts with distinct morphologies. Moreover, many of the previous synthesized Pt dependent NPs with hollow, cage, and frame structures are homogeneous cubic, octahedral, rhombic dodecahedral, etc. But reports on the fabrication of shape controlled with highly compositional segregated features have not been considered more. Moreover, it should be pointed out that in the previous reported analysis, compositional segregated Pt based nanomaterials have a deficiency in structure control, which is vital for the fabrication of noble metal NFs with more advanced applications. However, the synthesis of highly compositional segregated NFs with complex structures is still challenging. For the first time, Chen et al. fabricated rhombic dodecahedra Pt_3_Ni NFs with 2 nm thin edges from PtNi_3_ polyhedron by the erosion of Ni followed by annealing. In this strategy, etching was conducted by dissolved oxygen present in air instead of harsh etchants or applied voltage. The open frame structure with surface crystallinity, segregated Pt-skin and 3D structure augmented the availability of the reactants for both inner and outer surfaces. As compared to commercial Pt/C, the open framework design displayed the phase segregation Pt skin structure, which exhibited a 36-fold enhancement in terms of ORR (Chen et al., 2014).

**Figure 3:**
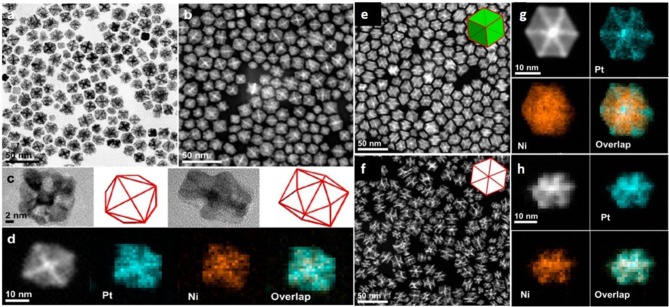
Morphological and structural characterizations for THH Pt-Ni NFs, RDH Pt-Ni NCs, and RDH Pt-Ni NFs. **(a)** TEM image, **(b)** HAADF-STEM image, **(c)** TEM images and corresponding geometric models of THH Pt-Ni NFs oriented along two typical projections, and **(d)** HAADF-STEM-EDX elemental mapping results for individual THH Pt-Ni NF. **(e)** HAADF-STEM image and **(f)** HAADF-STEM-EDX elemental mapping results for individual RDH Pt–Ni NC. **(g)** HAADF-STEM image and **(h)** HAADF-STEM-EDX elemental mapping results for individual RDH Pt-Ni NF. Modified with permission from Ding et al. (2016) Copyright 2016, American Chemical Society.

Another group has explained an effective procedure for the synthesis of tetrahexahedral PtNi NCS (THH PtNi NCs) as well as rhombic dodecahedral PtNi NCs (RDH PtNi NCs) with more segregated features (Ding et al., 2016). It has been reported for the first time that THH PtNi NCs have been gained in a simple organic solution system. These THH PtNi NCs as well as RDH PtNi NCS were simply obtained by tuning the ratio of oleylamine and oleic acid while keeping the other conditions constant. The synthetic procedure demonstrated that the use of dodecyltrimethylammonium chloride (DTAC) as well as changing the ratio of oleylamine, oleic acid plays important part for the synthesis of THH PtNi NCs and RDH PtNi NCs. However, by simple acetic acid treatment and removal of Ni template, the THH and RDH PtNi NFs were obtained. Due to their highly compositional segregated design, these solid PtNi NCs were transformed into the PtNi NFs which showed the more open feature. These PtNi NFs presented higher performance toward ORR and for alcohol oxidation due to enhanced consumption of Pt (Figure 3). Another group has proposed the synthesis of tetrahexahedral (THH) PtNi NFs by the removal of the Ni component of tetrahexahedral (THH) PtNi NPs by using carbon monoxide (CO). They demonstrated that the Ni was removed from the [100] direction by using CO molecules. However, these Pt_3_Ni NFs possess a 3D open structure, high index facets and thin segregated Pt layer. Furthermore, Co could be produced from the carbon support through thermal treatment in the presence of oxygen. This approach could be used for the preparation of industrial level nanocatalysts (Wang et al., 2017a). In the same way, Luan et al. also fabricated Pt-Ni Tetrahexahedral NFs by Co etching.

Chen et al. (2019) studied the reaction procedure of seeded co-reduction for the fabrication of PtNi seeded core frame structures. By controlling the shape of seeds, rhombic dodecahedra structures were obtained by depositing the Pt on their corners and edges. Different Pt salts and the ratio of Pt and Ni salts affected the shapes of seeds as well as the final morphology. Moreover, the Ni core of these hierarchical nanoarchitectures could be etched by the acetic acid which forms the concave structure and finally hollow NFs. In addition, the morphology and composition of the product could be manipulated by controlling the rates of etching and interdiffusion between two metals. The final Pt_3_Ni NFs are more stable in acidic environments and show distinct electronic properties that are different from the primary core frame nanoarchitectures because of the alloying of Pt with Ni. However, this seeded co-reduction strategy can be extended for the fabrication of other Pt-based complex core frame rhombic dodecahedral NPs. Moreover, hollow NFs used low acetic acid etching, which provides a simple means to change the phase segregation of bimetallic polyhedra for hollowing out the alloyed structure with more unique morphologies. Stamenkovic et al. explained that the 3D Pt anisotropy of PtNi rhombic dodecahedra can be controlled by changing the ratio between Pt or Ni salts; as a result, hollow, and excavated NFs can be gained after Ni corrosion. In excavated NFs, at the interior of excavated NFs Pt rich sheets were extended while hollow NFs had voids inside Pt rich edges. The excavated NFs had higher ORR performance which was due to the higher Ni amount in the near surface and the stretched 2D sheet-like structure within the NFs, which exposed Pt sites (Becknell et al., 2017).

In another example, Pt was coated on the surface of Cu nanocubes in order to synthesize the rhombic dodecahedral NFs (RD NFs). Moreover, growth in the [100] direction of Cu nanocubes leads to the RD. However, with the addition of Pt salts [100], vertices of Cu-CuPt rhombic dodecahedra NCs could be stretched to the spiny CuPt RD NCs. By removing the Cu content from the seed, CuPt RD as well as spiny CuPt RD NFs were gained. Because of their open frame design, both of these NFs showed a higher catalytic performance (Lyu et al., 2017).

**Figure 4:**
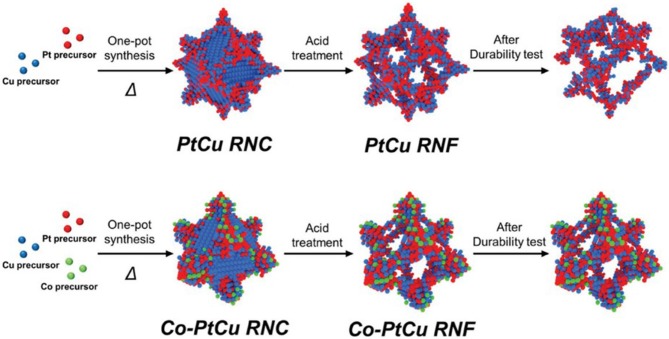
Schematic illustration for the formation process of Cu@PtCu rhombic dodecahedral nanocrystals (PtCu RNC), PtCu rhombic dodecahedral nanoframes (PtCu RNF), Co-doped Cu@PtCu rhombic dodecahedral nanocrystals (Co-PtCu RNC), and Co-doped PtCu rhombic dodecahedral nano-frames (Co-PtCu RNF). Adapted with permission from Kwon et al. (2018b) Copyright 2018, WILEY-VCH Verlag GmbH & Co. KGaA, Weinheim.

The additional component to the binary catalyst serves as an efficient and robust means of structural as well as compositional stability of electrocatalyst. Kwon et al. (2018b) reported that the insertion of Co to PtCu alloy NFs gives ternary PtCuCo rhombic dodecahedral NFs along the reinforced vertex of PtCuCo ternary NFs. The Pt deposited on the edges of the Cu template leads to the formation of PtCu alloy, and then this PtCu alloy enables the *in situ* decay of the third constituent, i.e., Co that penetrates into the PtCu segment to make the desired PtCuCo phase. Furthermore, the final Co-doped PtCu rhombic dodecahedral NFs (Co-PtCu rhombic nanoframe) display the vertex strengthened structure, suggesting that the deposition of Co-occurs in the region of the vertices of PtCu NFs (Figure 4). Similarly, Huang et al. (2018) fabricated the PtCuNi rhombic dodecahedra NFs (NFs) by a two-step etching method. Firstly, PtCuNi concave rhombic dodecahedra formed by oxidative etchant and then PtCuNi NFs formed through the acid etchant. By controlling the reaction time and Ni precursors, PtCuNi solid rhombic dodecahedra seeds' shape can be controlled. To investigate the evolution of PtCuNi NF, atomic ratios, particle size, and morphology of ternary Nfs are precisely examined. In another report, Ye and co-workers (Ye et al., 2017) synthesized the Pt_4_PdCu_0.4_ NFs by the Cu-assisted deposition etching process, and these NFs showed improved electrocatalytic properties due to structural and electronic effects.

### One Pot Etching

Using pre-synthesized templates like Pd or Ag NPs in the synthesis of hollow nanostructures has some limitations. (1) The synthetic procedure requires more steps such as template synthesis, shell growth as well as template removal, which limits practical use. (2) Most of the synthetic procedures utilize pre-synthesized templates, which are based on the noble metals like Pd or Ag as sacrificial template and waste expensive materials. (3) The resultant shape of hollow nanocages and NF structure is limited to the initial shape of the pre-synthesized templates. So, in order to overcome these disadvantages, for the tuning of surface energies, nanoscale phase mixing or segregation, *in situ* template formation has been proposed for the fabrication of novel hollow NCs. For the synthesis of a wide variety of binary and multimetallic NPs with more defined morphology, one pot synthesis is more preferable. Moreover, binary NCs are metastable, allowing the synthesis of NCs *in situ* phase segregation or even the removal of template leads to the hollow nanoarchitectures to be accomplished in one step.

**Figure 5:**
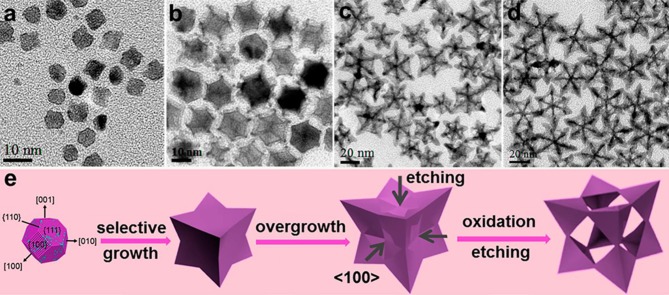
Representative TEM images of the intermediates obtained at different reaction time during the synthesis of concave PtCu_2_ octopod nanoframes: **(a)** 0.5, **(b)** 1, **(c)** 12, and **(d)** 18 h, respectively. **(e)** Schematic of the major steps involved in the formation of PtCu_2_ CONFs. Adapted with permission from Luo and Shen (2017) Copyright 2017, American Chemical Society.

Carbon corrosion and leaching of transition metals during electrochemical cycling may degrade the catalyst and may decrease its durability. To overcome this problem, an active and stable catalyst is needed. Currently, polyhedral PtNi NFs have been fabricated and show the promising electrocatalytic performance for fuel cells. However, these NF catalysts have been mostly developed by the multi-step synthetic procedure, which made cost saving massive production a big challenge toward practical applications. So, engineering of self-supported as well as high-performance NF catalysts through one pot procedure is of great importance. To synthesize stable and active catalysts, Luo and Shen described a one-pot procedure for the construction of concave PtCu_2_ octopod NFs (CONFs) with higher index facets in which CTAB is used as a structure directing agent. The CONF nanoarchitectures meet the critical design requirements for more efficient nanocatalyst-like highly open nanoarchitectures with 3D catalytic surface and a stepped surface composed of multiple index facets which showed enhanced catalytic activities. The self-supported CONFs catalyst removed the carbon erosion and facilitated the reaction by enhanced mass transport. Furthermore, CONFs are assembled by the eight symmetry feet that showed excellent physical and electrochemical stability. The evolution mechanism indicates that the octopod NFs formed from solid octapods by sequential erosion, and the overgrowth from concave cubic structure to concave octopods is comparatively slow (Figure 5) (Luo and Shen, 2017).

By using the one pot solvothermal strategy, Huang et al. (2018) reported 3D PtCu alloy nanocages with an open structure, large exterior and interior surfaces that are accessible for the reactants. Arginine and cetyltrimethylammonium chloride (CTAC) performed as the co-structure directing agent as well as the stabilizer. In this method, Cl^−^/O_2_ and oleylamine served as the etchant and co-reductant, respectively. These frame-like nanocatalysts showed greater activity and stability.

### Structurally Fortified Nanoframes

More definite 3D NF design displays well-defined features like corners, edges, and facets but they degrade in harsh conditions (Park et al., 2018). However, the catalytic active surface could be preserved by the fortification of the most vulnerable sites, such as vertices through alloying and heterostructure formation, and the insertion of supported materials into NFs to maintain the overall higher surface area of the structure without losing the surface area of the exposed sites. For the formation of NFs, higher surface energies and vertices of NFs are vulnerable to the structural degradation in the chemical etching process as well as in electrocycling tests. Many groups reported the synthetic procedure in order to reduce the vulnerability of vertices by the growth of additional metals on the active vertices, which results in hetronanostructure formation at the vertices or at the alloying of vertices. However, sometimes etching and galvanic replacement reaction could take place in one process. For instance, Wu and co-workers grew Au islands on vertices of PtNi NFs. They used a protocol based on the selective galvanic replacement reaction and preferred etching, in which the role of etching was more obvious for NF synthesis. The etching was regulated by dimethylglyoxime as Pt was stable to dimethylglyoxime, therefore NFs with protected Pt edges were obtained even after etching. During MOR, the overgrowth Au sites impeded the structural deformation of PtNi NFs and the catalytic activity of Pt_3_Ni NFs with the 10% Au remains unchanged after the 3,000 cycles.

### Insertion of Structural Support Into the Nanoframes

**Figure 6:**
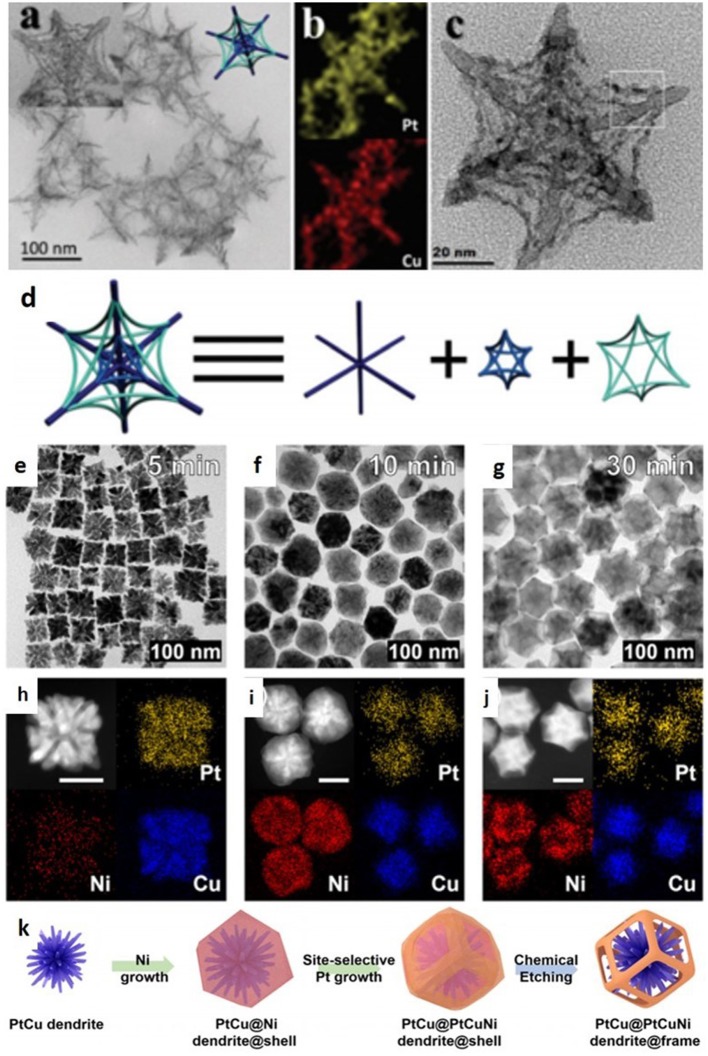
**(a)** TEM images of hexapod backbones with thin stretchers (thinHBS). **(b)** Element mapping results of thinHBS. **(c)** HRTEM image of thinHBS. **(d)** The anatomic structure of thinHBS. Modified with permission from Nosheen et al. (2016) Copyright 2016, The Royal Society of Chemistry. **(e–g)** Temporal TEM images and **(h,i)** elemental mapping analysis obtained at **(e,h)** 5 min, **(f,i)** 10 min, and **(g,j)** 30 min. Scale bars in **(h–j)** are 30 nm. **(k)** Schematic illustration of formation of PC@PCN dendrite@frame. Modified with permission from Park et al. (2017a) Copyright 2017, American Chemical Society.

The inherently weak NFs can be brutally damaged when subjected to long cycling time in electrochemical reaction, due to which a reduction in electrocatlytic performance may be observed. Hence, fabrication of NFs with strong structure support is most important and challenging. We have synthesized PtCu ultrathin NFs by the one-pot method, and these ultrathin NFs were abbreviated as hexapod backbones with thin stretchers (thinHBS). In addition to thinHBS, some other partially etched complex polyhedral hollow structures have also been synthesized. CuCl_2_ and NaI were responsible for etching to modify the shape, whereas ethanolamine, and amino acids stabilized the [111] the domains. The combination of growth and the etching process smoothly controlled the shape. The transmission electron microscope image showed a two-dimensional picture which looks exactly like hexagonal snowflakes, and the adjacent trunks have a few stretchers which are attached to the trunks symmetrically. The trunk length was found to be 40 nm, the diameter was 5 nm and thickness of the stretchers was about 1.3 nm. The synthesized structures may be beneficial for electrocatlytic applications with enhanced activity and stability owing to ultrathin stretchers and internal trunks which provide support for stability (Figures 6a–d).

Moreover, trimetallic catalysts are more active and stable than bimetal alloys. In a recent study, Kwon et al. (2018a) explained the idea of a dendrite@frame-like nanostructure in which the outer PtCuNi NFs are supported by inner PtCu dendrite. Moreover, branches network as well as attached joints can reinforce the multiframe structure and could maintain the same structure during harsh conditions. In order to boost the consumption of Pt, it is necessary to have a more active PtNi alloy part for both the interior support as well as for the outer multiframes. Furthermore, the presence of nanopores in multiframes can be advantageous to entrap the gaseous reactant into nano-confined space and can show higher catalytic performance. Burst reduction and deposition of Ni after the PtNi alloying/dealloying created multiple PtNi bridges among the PtNi and superimposed interfused multiframes. By the Ni erosion, PtNi multiframes with embedded PtNi dendrites of 72 nm were gained. However, these PtNi multiframes showed a higher electrochemical active surface area and ORR performance owing to porous, more active sites, and higher index facets. Similarly, Park et al. (2017a) fabricated firm robust PtCuNi NFs, supported with an interior PtCu dendritic structure. In the formation process, first a PtCu cubic dendritic structure was formed, which evolved into PtCu@Ni dendrite@shell structures after constant deposition of Pt on the surface, and after etching process, PtCu@PtCuNi dendrite@frame (PC@PCN) formed. The detailed process showed that the morphological development into rhombic dodecahedral NCs requires Pt for the growing Ni layer (Figures 6e–k).

### Other Uncommon Methods

In addition to the above-mentioned methodologies, a cage/frame nanostructure has been developed by the other synthetic procedures such as control decomposition kinetics, long chain alcohol (Jeyadevan et al., 2014), Ostwald ripening (Zhang et al., 2018a), etc.

### Nanoframes Other Than Pt and Pd Metals

Noble metal NFs other than Pt and Pd, including gold (Au), ruthenium (Ru), iridium (Ir), and silver (Ag), have also been synthesized, but these NFs commonly have not been used for electrochemical applications in fuel cells. For their synthesis, etching, the galvanic replacement reaction and Kirkendall effect strategies were used. To cover the NFs' synthesis for all types of noble metals, we have also included a short description of noble metal NFs other than Pt and Pd.

Ultrathin triangular gold NFs have been synthesized through the etching process by Xue's group. In this strategy, Ag nanoprisms were used as the sacrificial template and HAuCl_4_ was continuously introduced to the solution for the deposition of gold on the Ag nanoprisms. Hydroxylamine was used as the reducing agent, and after deposition of gold on the surface of Ag prism, etchants (H_2_O_2_+NH_4_OH) were used to completely remove the Ag prism for the synthesis of ultrathin NF. H_2_O_2_ oxidized the Ag atoms and NH_4_OH prevented the formation of other particles like AgOH and Ag_2_O by dissolving the oxidized silver. Etchants were slowly added into the solution because their rapid introduction may destroy the frame structure (Shahjamali et al., 2013). In another example, ultrathin Au NFs with a thickness of 1.6 nm have been synthesized by using the Ag template with decahedral shape, twinned pentagonal rods, and icosahedra to fabricate analogous NFs. H_2_O_2_ was used as an etchant for the dissolution of Ag (McEachran et al., 2011).

Rhodium cubic NFs have been synthesized by Xia and co-workers, in which Pd nanocubes have been used as seeds and deposition of Rh at the edges-corners of Pd seeds occurred due to the adsorption of Br^−^on facets and converted them into bimetallic Pd-Rh core-frame concave cubes. By selective etching of Pd and continuous growth of Rh, they were converted into Rh NFs (Xie et al., 2012). Another group reported the fabrication of Ru NFs by using truncated octahedal seeds, which are converted to PdRu core-frame octahedral structure that are finally converted into Ru octahedral NFs with 2 nm thick frame walls by the selective dissolution of the Pd core by etching (Ye et al., 2016). Very recently, the same group also synthesized Ru cuboctahedral NFs with the face centered cubic (fcc) phase and walls that were a few atomic layers thick by a combination of etching and the galvanic replacement method (Zhao et al., 2019). In addition to monometallic nanoframes, bimetallic nanoframes of Au, Ag, Ru, and Ir have also been synthesized. For instance, Ag/Au nanoshells with voids and pores have been fabricated by utilizing silver NPs as sacrificial templates in the presence of H_2_O_2_ as an etchant (Wu et al., 2012). Zhang's group fabricated Rh-based RhCu nanooctahedron frames/C, RhPdCu nanopolyhedral frames/C, and RhNi porous nanopolyhedral frames/C by using 3d metals like Cu and Ni as the sacrificial template by selective etching (Zhang et al., 2015b).

Ir-based multi-metallic double layer NFs (DNF) with NF@NF structure were developed by Lee et al. By controlling the kinetics of two types of iridium salts and salts of transition metals like Ni and Cu, firstly core shell alloy@alloy formed in one step and then these structures converted into rhombic dodecahedral IrNiCu DNF by preferential etching. Single-metal NFs formed by the addition of only one type of Ir salt, which showed that for the formation of double layer NFs, two kinds of Ir-metal precursors are important. The structure of Ir-based NCs have also been converted to other double layer NF morphologies, including octahedral NFs and CuNi@Ir core-shell structures, by changing the experiment conditions. The synthesized IrNiCu double layer NFs have been used for an oxygen evolution reaction (OER) in acid medium, which showed enhanced activity as compared to commercial Ir/C. These NFs also showed good stability toward OER that could be due to the frame which stops the growth and aggregation of NPs (Park et al., 2017b).

Galvanic replacement reaction has also been used for noble metals other than Pt and Pd by many groups to synthesize monometallic and bimetallic hollow structures, nanocages, and NFs of different morphologies by using an Ag template of desired shapes (Zeng et al., 2009; Lai et al., 2015). Colloidal gold nanorings (Au NRs) with high monodispersity were synthesized with the one pot method by galvanic replacement by Du and Wang. PVP was used as coating on the cobalt nanoparticles, and then these PVP-coated nanoparticles generated a one-dimensional assemblage of cobalt nanoparticle chains which were used as the sacrificial template and galvanically replaced by Au to form Au NRs (Hu et al., 2014). Li's group reported single crystalline octahedral AuAg alloy nanoframes by the one pot galvanic method in which polyhedral truncated Ag NPs formed in solution (Hong et al., 2012b). The same group has also developed Ru-Cu nanocages and core-shell Cu@Ru NCs by a one-step galvanic reaction (Chen et al., 2016).

An Au@Ag core-shell, Au@void@AuAg nanoshell, and Au@void@Au NF have been reported by Neretina's group. Au@Ag core-shell structures produced a noble metal rattle shape by galvanic reaction where a moveable core was present in the hollow shell. Gold, platinum and palladium templates of Wulff structures were firstly converted into core/shell structures by the reduction of silver ions, which were further converted by the replacement of silver with gold.

The Wulff-shape core could be restricted in a nanoshell/cage/frame by adjusting the shape of the shell, epitaxial correlation to substrate, and the replaced amount of shell. These structures could be considered important as there is a precise gap between the core and shell. The internal side of these structures consist of the core, shell as well as substrate, which may offer good functional potential. Furthermore, the core present in the shell may provide protection and it is also linked with the surrounding reactants due to a porous cage or frame, which may be beneficial for many applications (Hajfathalian et al., 2016). González's group used a sequential galvanic replacement reaction and the Kirkendall effect at room temperature to fabricate double-walled AuAg hollow nanoboxes (González et al., 2011).

In addition to the above-mentioned methods, some other methods have also been used to synthesize hollow structures. Hollow rutehenium (Ru) octahedral (Oh) nanocages have been developed by *in situ* formed metastable copper octahedrons. Core shell Cu-Ru NPs with meta stability formed by the co-decomposition of copper and ruthenium precursors formed, and a consequent dissolving of copper core dissolution of the core Cu produced hollow octahedron shaped Cu-doped Ru nanocages. These frame structures were also used as electrocatalysts for OER (Yoon et al., 2014). Gu et al. fabricated AuAg shells and porous AuAg NFs by using citrate capped silver nanoparticles by the galvanic replacement reaction. The AuAg nanoshells formed in the presence of AuBr^2−^ and porous AuAg NFs formed in the presence of AuCl^4−^. The AuBr^2−^ was also used to synthesize Au@AuAg rattles (Bai et al., 2015). Qin's group reported a method to synthesize Ag@AgAu core-frame cubic structures by the co-reduction of two metals in the presence of ascorbic acid (Sun and Qin, 2015).

### Electrochemical Properties

In electrocatalytic reactions, there is contact between the reaction and active sites of metal. This interaction is due to various energy levels of the outermost electrons, and the electron transfer takes place to reduce the overall energy of the system. The procedure is generally completed by bond breaking and formation, which include chemical sorption and generation of intermediate species. The metals' d band center in relation to the Fermi level is considered a significant tool for measuring the power of interaction with reaction species. The d band center of the metals could be modified by fabricating alloy nanostructures or heterogeneous atomic layers, which could improve the electrocatalytic properties. The shifts in d band center values could be ascribed to the transfer of charge or lattice change owing to the addition of more than one metal. In case of alloying, the enhancement in electrocatalytic properties could be credited to charge conversion from the transition metal atoms of less electronegativity to the more electronegative platinum atoms, and another reason is the shortening of the platinum-platinum bond distance, known as compressive strain. It may happen due to the variations in elements size of different metals. However, the charge transfer as well as strain may cause the downshifting of d-electronic states of platinum surface atoms relative to the Fermi level of alloys, and hence may reduce the poisoning effect of the oxygen reduction's intermediate species.

Moreover, it has been observed that the occurrence of different kinds of transition metals in the alloy nanostructures provide a chance for maneuvering O_2_ species on different surface sites and facilitate an additional bi-functional process to accelerate the oxygen reduction kinetics. For improved electrocatalytic performance, alloy NFs with platinum-skin, platinum-skeleton, or frame have been developed as promising candidates. For example, Han and co-workers have synthesized a Pt-Cu alloy octahedral Nfs (PtCu AONFs) having spiny nano-thorns on the tops by the one-step method. The insertion of nickel ions was crucial for NF formation. The synthesized PtCu AONFs showed enhanced activity and stability for ORR. The mass and specific activities were 2.8 and 2.6 times that of commercial platinum-carbon catalysts (Zhu et al., 2019). Furthermore, these PtCu AONFs also showed better results for MOR than Pt/C. In case of PtCu alloy, the surface electronic structure of pure platinum was modified and the electrocatalytic properties enhanced toward oxygen reduction reaction because of a drop of adsorption energy between platinum and oxygen species.

### Effect of Surfactants on Electrochemical Properties

For the shape control of nanoparticles, surfactants are usually used, which may also be adsorbed on the exposed crystal facets and thus affect their electrochemical performances. In short, the surface covered with surfactant may suppress the catalyst performance. To overcome this problem, removal of surfactant is necessary. Until now, different kinds of treatments have been developed for the removal of surfactants. First, a simple washing procedure is performed, followed by separation through centrifugation, and then the following treatments:

- Thermal annealing: The catalysts are heated in furnace at high temperature in air.
- Chemical washing: The catalysts are dispersed in acetic acid for a suitable time, followed by washing through centrifugation to remove unwanted impurities.
- UV-Ozone treatment. Catalyst is deposited on the glassy carbon electrode, which is then irradiated with UV-Ozone to remove the surfactants after drying in air.
- Electrochemical cyclic voltammetry is used to remove the surfactants covered on the surface of the catalysts.

## Applications in Fuel Cells

### Liquid Fuel Oxidation Reaction

In the polyelectrolyte fuel membrane cells (PEMFCS), gaseous hydrogen fuel is replaced by liquid fuels like methanol in direct methanol fuel cells (DMFC), formic acid in the direct formic acid fuel cells (DFAFC) and ethanol in direct ethanol fuel cells (DMFC) (Park et al., 2017a). For the small portable electronic devices, liquid fuels are more suitable because of their easily exchangeable fuel cartridges, higher energy density as well as faster charging rate. Because of their higher number of advantages, which are described above, the use of liquid FCs presents more challenges. The main issue is the partial oxidation of liquid fuels which leads to the sluggish oxidation reaction kinetics, and it causes a reduction in the number of transferred electrons. In DMFC, <30% of the energy can be exploited as electricity. Another major issue is the crossover of fuel molecules from anode to cathode through diffusion by the proton exchange membrane in the cell. Due to the crossover mechanism, it not only reduces the overall cell voltage but also contaminates the cathode catalyst. Due to the partial oxidation of liquid fuel, it forms CO which adsorbs on the surface of the cathode catalyst and effects the ORR performance of the cathode in cell operation. This procedure is called CO poisoning. However, Pt-based alloys are more familiar, tolerate CO poisoning and show the excellent catalytic performance. Mostly, previous studies explained the strategies to synthesize the hollow nanoarchitectures which showed higher electrocatalytic properties.

In addition to the above electroxidation reactions, the hydrogen oxidation reaction (HOR) is also an important reaction in fuel cells. HOR is a rapid one-electron oxidation route that occurs at anode. Until now, noble metals like Pt-, Pd-, Ir-, and Rh-based monometallic and alloy structures have been used as electrocatalysts for HOR, but currently no paper is available on noble metal nanoframes, which is why we will not focus on HOR applications in fuel cells. For deeper understanding of HOR, the reader is directed to related review papers (Cong et al., 2018; Davydova et al., 2018).

### Formic Acid Oxidation Reaction (FAOR)

DFAFCs are the devices which catalyze the reactions among formic acid at the anode and O_2_ at the cathode and change the chemical energy into electricity (Ye et al., 2017). In recent decades, for the energy source formic acid is usually used as a fuel in DFAFCs, which have received more attention. Moreover, formic acid is liquid at room temperature and is the most promising fuel. It replaced molecular hydrogen and assisted storage as well as transportation. DFAFCs show higher power density, faster oxidation rate, and higher cell potential with mild fuel crossover.

In case of FAOR, the main limitation for the Pt catalyst is struggle among the direct dehydrogenation path and indirect oxidation path, which produce CO_2_ directly and CO as an intermediate, respectively. So, enhancement of the direct pathway is necessary to assist the detachment of CO from the surface. In addition, surface as well as electronic structure play significant roles in the half reaction. However, separation of Pt atoms with others not only suppresses the indirect path but is also helpful for the CO stripping from the platinum surface. In case of alloys, atomic separation is gained, which not only tailors the surface electron densities but also stimulates direct dehydrogenation. However, synthesis of stepped atoms on the surface provides additional chance for enhanced CO stripping. Thus, fabrication of open structures and alloy formation are vital for efficient and durable catalysts for FAOR.

**Figure 7:**
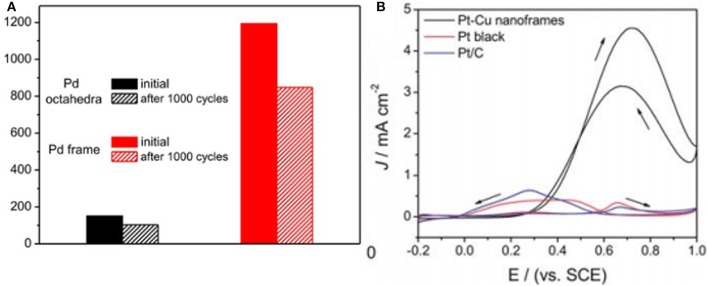
**(A)** Electrochemical durability of the Pd octahedral nanoframes and solid Pd octahedra catalysts. The current density was normalized against the corresponding ECSA. Modified with permission from Wang et al. (2016) Copyright 2017, American Chemical Society. **(B)** Cyclic voltammetric CV profiles of octahedral Pt-Cu nanoframes in a 0.25 M HCOOH + 0.5 M H_2_SO_4_ solution (50 mV s^−1^). The specific current density (J) was normalized to the electrochemically active surface area (ECSA). Modified with permission from Nosheen et al. (2013) Copyright 2013, Royal Society of Chemistry.

Wang et al. fabricated Pd NFs with more defined morphology by directly excavating the solid NCs. However, in comparison with the solid Pd catalyst, these NFs display good activity as well as durability toward FAOR because of their 3D open structure. Pd NFs displayed high activity in comparison to the Pd octahedra catalyst and a 7.5-fold increase for peak current. Because of their 3D open structure, there is a larger fraction of edges and corner atoms, which lead to good catalytic performance in terms of FAO. Moreover, the long-term stability of catalysts is estimated by the durability test as depicted in Figure 7A. It is noted that the nanostructure of Pd NFs were balanced and showed good stability after 1,000 cycles of durability tests (Wang et al., 2017b).

In another report, Nosheen et al. (2013) used the one pot method for the fabrication of single crystalline octahedral PtCu NFs in an aqueous system. When compared with the platinum black and platinum/carbon catalyst, these octahedral PtCu NFs showed good specific activity toward FAOR due to their exclusive frame structure and combined effect of Pt and Cu (Figure 7B). Furthermore, comparison of electrocatalytic properties of Pt and Pd-based nanoframes toward FAOR, MOR, EOR, ORR, and bifunctional electrocatalysis is given in Table 1 for better understanding.

**Table 1:** Comparison of electrocatalytic properties of Pt and Pd-based nanoframes toward FAOR, MOR, EOR, ORR, and bifunctional electrocatalysis.

**Electrolyte**	**Types of electrocatalyst**	^*^ECSA (H_**upd**_)	^*^Mass activity	^*^Mass activity enhancement vs. Pt/C	**Type of electro-chemical reaction**	^*^Specific activity	**Specific activity enhancement vs. Pt/C**	**Stability (%) vs. Pt/C**	**Reference**
0.5 M HCOOH + 0.5 M HClO_4_	Pd NFs	_	_	7.5^a^	FAOR	_	_	80^b^	Wang et al., 2017b
0.25 M HCOOH+0.5 M H_2_SO_4_	Octaherdral PtCu NFs	4.5	_	_	FAOR	_	4.55^c^	_	Nosheen et al., 2013
0.5 M H_2_SO_4_ +1 M CH_3_OH	PtCu_2_ CONFs, PtCu UONFs	44.552.1	_	3355.6	MOR	155.9	7.5	7253.9	Luo and Shen, 2017
0.5 M H_2_SO_4_+2 M CH_3_OH	Pt-Cu yolk cage alloy NCs	8.3	0.7	_	MOR	_	2.8^c^	_	Zhang et al., 2013
0.1 M HClO_4_ + 1.0 M CH_3_OH	Pt-Cu NFs	41.3	0.98	1.72^c^	MOR	2.35	2.77^c^	_	Ding et al., 2015
0.1 M HClO_4_ +1 M CH_3_OH	PtCu_3_ nanocages	35.7	_	_	MOR	_	14.1^d^	_	Xia et al., 2012
0.5 M KOH+1 M CH_3_OH	PdCu Alloy Flower-like Nanocages	22.1	823	2.7^e^	MOR	_	_	_	Chen et al., 2018
0.5 M KOH + 1 M ethanol	Pt_4_Ni NFs, PtNi_4_ porous octahedra	_	_	4.54.1	EOR	_	5.90 5.50	**_**	Wang et al., 2015b
0.1 M HClO_4_	Pt cubic NFs	_	0.45	0.82	ORR	0.53	1.17	140	Park et al., 2016
0.1 M HClO_4_	PdPt NCs Octahedral nanocages (ONCs), Cubic nanocages (CNCs), Octahedral dendritic hollow (ODH) NCs, Cubic dendritic hollow (CDH) NCs	40.342.341.539.8	764.7 178.1 393.9 324.4	2.67^f^1.39^g^1.38^f^2.52^g^	ORR	_	_	_	Hong et al., 2012a
0.1 M HClO_4_	Cubic nanocage Octahedral nanocage	46.838.2	_	0.75	ORR	_	1.98	36 ^j^	Zhang et al., 2015a
0.1 M HClO_4_	Ultrathin icosahedral Pt-enriched nanocages, octahedral nanocage, cubic nanocages	45.0	_	1.12^h^0.750.38	ORR	_	2.48^h^ 1.98 0.82	_	He et al., 2016
0.1 M HClO_4_	Pt-Based Icosahedral Nanocages, Pt-based octahedral nanocages	36.5	_	_	ORR	_	3.50^i^ 1.98	18^j^	Wang et al., 2016
0.1 M KOH	Cu-Pt nanocage	68	_	0.32	ORR	_	0.47	_	Dhavale and Kurungot, 2015
0.1 M HClO_4_	Alloy Cu_3_Pt NFs	30.2	_	_	ORR	_	_	_	Han et al., 2014
0.5 M KOH	PtCu hollow cubic NFs	23.4	_	0.732	ORR	_	3.12	19	Wang et al., 2018a
0.1 M HClO_4_	Spiny rhombic dodecahedral CuPt NFs, CuPt alloy RD NFs	39.0953.87	_	0.580.81	ORR	_	2.19	24.7	Lyu et al., 2017
0.5 M KOH	Cu-rich rhombic dodecahedral PtCu NFs	19.18	_	1.4^k^	ORR	_	2.7^k^	21.2	Huang et al., 2019
0.1 M HClO_4_	Pt_3_Ni NFs	1.52	_	0.9	ORR	_	1.5	40	Chen et al., 2014
0.1 M HClO_4_	Pt-Ni multiframes	73.4	_	1.51	ORR	7.06	2.09	6.23	Kwon et al., 2018a
0.1 M HClO_4_	Excavated NFs Hollow NFs	48.160.2	_	0.60.3	ORR	_	1.35^l^ 0.55	_	Becknell et al., 2017
0.1M HClO_4_	PtCuNi rhombic dodecahedra NFs	_	_	0.86	ORR	_	1.65	57	Huang et al., 2018
0.1 M HClO_4_	PtPdRu dendritic nanocages	96.8	_	2.61^m^	ORR	_	1.81^m^	87	Eid et al., 2015
0.1 M HClO_4_	PtPdNi truncated octahedral nanocages	55.7	_	1.14^n^	ORR	_	1.52^n^	85	Wang et al., 2019
M KOH0.5 M KOH+1 M CH_3_OH	PtCu NFs	12.4	_	0.2112.26	ORR MOR	_	1.71 18.2	64.4	Zhang et al., 2016
0.1 M HClO_4_0.1 M HClO_4_ + 1 M CH_3_OH	Co-PtCu rhombic dodecahedral NFs	31	_	1.56°4.11°	ORR MOR	_	5.03^o^ 13.3°	73.163.5	Kwon et al., 2018b
0.1M HClO_4_, 0.1M HClO_4_ + 0.1M HCOOH	Pt_4_PdCu_0.4_ Alloy NFs	_	_	1.04	ORR FAO	_	2.41 8.70	9.210.2	Kwon et al., 2018b
sM HClO_4_	Pt_3_Ni alloy tetrahexahedral NFs	_	_	0.474.2	ORR HCOOH	_	2.5	_	Wang et al., 2017a
0.5 M H_2_SO_4_+2 M CH_3_OH0.25M HCOOH + 0.5 M H_2_SO_4_	Pt-Pd-Rh-Ag NFs	_	300	3	MOR HCOOH	_	2.8	_	Saleem et al., 2016

* *Units used for Pt based nanoframes are: ECSA (H_upd_) (m^2^${\text{g}}_{\text{Pt}}^{-1}$), mass activity (A ${\text{mg}}_{\text{Pt}}^{-1}$), specific activity (mA ${\text{cm}}_{\text{Pt}}^{-2}$) While in case of PtPd NCs electrochemical values were calculated and compared with both Pt and Pd content, so units used mA ${\text{mg}}_{\text{Pd}+\text{Pt}}^{-1}$, etc. In case of Pd based NCs, electrochemical values were calculated, and compared with Pd content, so units used ${\text{mAmg}}_{\text{Pd}}^{-1}$, etc*.

a *Compared with Pd octahedral catalyst (unit used: μA cm^−2^)*.

b *Compared with the pristine Pd octahedral NCs*.

c *Also compared with Pt black*.

d *Compared with PtCu_x_ nanoparticles and the commercial Pt electrocatalyst*.

e *Compared with commercial Pd/C*.

f *Compared with OD NCs*.

g *Compared with CD NCs*.

h *Also compared with Pd-Pt core-shell/C*.

i *Compare with Pt-based octahedral nanocages*.

j *Compared with the pristine Pt/C*.

k *Also compared with the Pt_18_Cu_82_ NCs, Pt_50_Cu_50_ NCS*.

l *Compared with hollow NFs*.

m *Also compared with PtPdRu nanodendrites*.

n *Also compared with Pd@PtPdNi MTOs*.

o *Also compared with PtCu RNF/C*.

### Methanol Oxidation Reaction (MOR)

**Figure 8:**
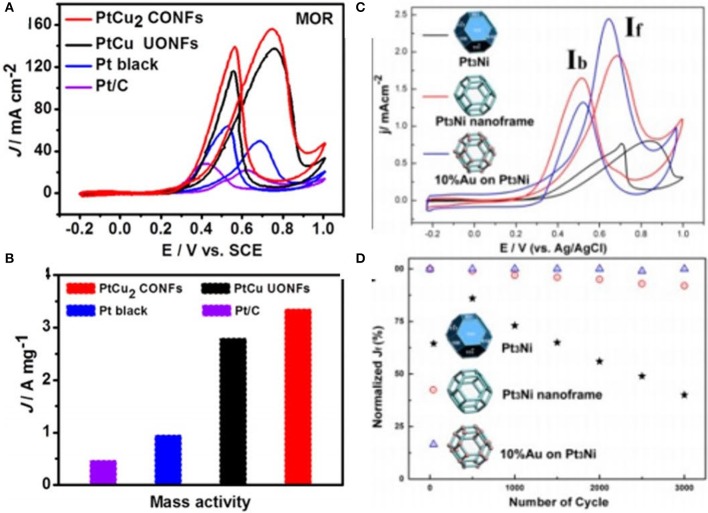
Cyclic voltammetry (CV) curves of different electrocatalysts for methanol oxidation reaction. **(A)** recorded at scanning rate of 50mV s -1 in 0.5M H2SO4+1M CH3OH solution, and formic acid oxidation reaction **(B)** Mass activity of PtCu2 CONFs, PtCu UONFs, Pt black, and Pt/C electrocatalysts for methanol oxidation reaction. Modified with permission from Luo and Shen (2017) Copyright 2017, American Chemical Society. **(C)** Cyclic voltammograms of methanol oxidation on different catalysts in 0.1M HClO4 containing 1M MeOH. **(D)** Loss of peak current density in a forward scan as a function of cycling numbers. Modified with permission from Wu et al. (2014) Copyright 2014, American Chemical Society.

In the case of DMFCs, MOR occurs at the anode with the multistep reaction, and it depends upon adsorption as well as oxidation of methanol on the catalyst. For the MOR, Pt is usually used as a catalyst due to its higher activity toward adsorption of methanol. In addition, pure Pt surface suffers from CO poisoning, which reduces the catalytic performance. Researchers have focused on the fabrication of Pt-based nanocatalysts, which not only reduces the CO poisoning but also displays good activity and stability. For instance, Luo and Shen (2017) fabricated the PtCu NFs along higher index facets by the one pot method, which showed good catalytic performance. In addition, by tuning the ratios of Pt as well as Cu precursors, two different octopod NFs like PtCu_2_ concave octopod NFs and ultrathin PtCu octopod NFs were synthesized. Because of their 3D surface area and higher index facets, these PtCu_2_ concave octopod NFs displayed good catalytic performance and more CO tolerance. Regarding MOR, these PtCu_2_ concave octopod NFs showed a 7-fold enhancement in activities as compared to the commercial Pt/C catalyst. However, in a severe environment of electrocatalytic reaction, these PtCu_2_ concave octopod NFs are more conserved as shown in Figures 8A,B. In another report (Zhang et al., 2013), PtCu nanocages were developed by using the glycine mediated reaction kinetics, which showed good activity and stability for the MOR in comparison to the Pt/C and Pt black catalysts. The current density value specific activity of PtCu yolk-cage nanoalloy was 2.8 mA cm^−2^, which was four- and 6-fold higher than Pt black and Pt/C, respectively. Similarly, Ding et al. fabricated rhombic dodecahedral PtCu NFs by using the one pot method. In comparison with the Pt/C as well as Pt black, these PtCu NFs presented good catalytic performance toward MOR due to their highly open nanostructure and synergetic effect among two metals (Ding et al., 2015). Moreover, Xia et al. (2012) synthesized the PtCu_3_ nanocages by the one pot method in organic solution. Toward MOR, these PtCu_3_ nanocages depicted excellent catalytic activity when compared to solid PtCu NPs as well as with single component Pt catalysts. The excellent catalytic performance was due to the special nanostructure as well as the synergetic effect among Pt as well as Cu. In addition to Pt metal, Pd alloy NFs have also been used for MOR. For instance, Chen et al. have synthesized the PdCu flower-like nanocage by using the galvanic reaction and disproportionation reaction, and in the synthetic procedure Cu_2_O octahedra were used as template. These PdCu flower shaped nanocages showed more catalytic activity in comparison with the Pd NPs in terms of MOR, which was 2.7 times greater than the commercial Pd/C. The higher catalytic properties were because of their large surface area and effect of Pd and Cu metals. In addition, these PdCu nanocages have shown good stability as well as excellent poison tolerance. For MOR, the presence of 10% Au on Pt_3_Ni NFs may increase the catalytic activity, and the structure was preserved even after 3,000 cycles. The Pt skinned surface of NFs that is formed via the etching process is the cause of enhanced durability, which prevented further etching of the Ni content. Furthermore, the addition of Au islands to the PtNi frame could fade the bindings of poisonous and adsorbed species on the surface, which may further enhance durability Figures 8C,D.

### Ethanol Oxidation Reaction (EOR)

**Figure 9:**
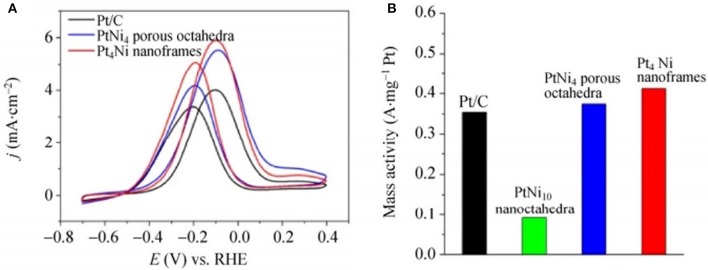
Cyclic voltammograms of commercial Pt/C, PtNi_4_ porous octahedra, and Pt_4_ Ni nanoframes in **(A)** 0.5 M KOH + 1 M ethanol (scan rate: 50 mVs^−1^); the current density was normalized to the ECSAs. **(B)** Mass activities measured at −0.1 V for ethanol oxidation on commercial Pt/C and three Pt-Ni catalysts; the current density was normalized to the weight of Pt loaded on the working electrode. Modified with permission from Wu et al. (2014) Copyright 2015, Tsinghua University Press and Springer-Verlag Berlin Heidelberg.

Ethanol is also a type of liquid fuel and used in direct ethanol fuel cells. For instance, Wang et al. developed 3D Pt_4_Ni NFs as well as PtNi_4_ porous octahedra by the corrosion of PtNi_10_ solid nanoctahedra and compared their ethanol electrooxidation under alkaline media. Current density values at the −0.1 V in the positive scan are the 5.50 and 5.90 mAcm^−2^ for the PtNi_4_ porous PtNi_4_ octahedra and Pt_4_Ni NFs which both display a higher value as compared to the commercial Pt/C value (4.01 mAcm^−2^). However, the corroded Pt_4_Ni NFs as well as the PtNi_4_ porous octahedra electrocatalyst were confirmed by mass activities, which are shown in the Figures 9A,B. The mass activity of Pt_4_Ni NFs exhibited an improvement factor of 4.5 vs. PtNi_10_ octahedra, and in the case of Pt_4_Ni porous octahedra the improvement factor was 4.1, which are much better as compared to the commercial Pt/C.

According to these analyses, it is proved that ethanol electrooxidation displays improved catalytic activities after corrosion from solid PtNi_10_ nanoctahedra to Pt_4_Ni NFs as well as PtNi_4_ porous octahedra. Among these, Pt_4_Ni NFs showed outstanding catalytic performance because of their higher surface area to volume ratio. In addition, PtNi_4_ porous octahedra, a transparent structure with more voids and Pt-rich surface, shows superior catalytic activity. That is why the corroded nanocatalyst is superior to parent NCs in which mostly Pt atoms are buried inside, as shown in Figure 9.

### Oxygen Reduction Reaction (ORR)

Pt-based nanocatalysts have been the most promising electrocatalysts toward ORR. But the main issue for platinum oxygen reduction is due to their lower abundance as well as higher price of Pt (Escudero-Escribano et al., 2018; Wang et al., 2018b). It is necessary to improve the efficacy of Pt atoms in catalysis by reducing Pt usage and to improve the ORR activity which is gained by both the surface and electronic structure. In terms of surface structure, hollow as well as open nanoarchitectures like nanocages and frames boost the number of Pt atoms on the edges and show higher activity for electrocatalysis. However, electronic effects act as another way for tuned ORR activity. As mentioned in the previous studies, the best method to tune the electronic structure is the alloying of Pt with other metals. In addition, Pt and Pd work differently, accumulate more electrons at Pt sites and may improve the catalytic performance in terms of ORR. However, noble metal-based NFs show higher catalytic activity and durability as compared to others. Park et al. (2016) reported the fabrication as well as catalytic activities of Pt cubic NFs with wall thickness <2 nm. The as synthesized Pt cubic NFs displayed a 6-fold enhancement in ORR mass activity over 20,000 cycles of repeated potential sweeping in comparison with Pt/C.

The morphology-based catalytic activities of several PdPt NCs in terms of ORR were studied, and their results have been compared with the commercial catalyst. For instance, Hong et al. synthesized nanocages of different shapes and compared them with the Pd@Pt core shell as well as NCs without hollow structure and commercial catalyst, and the as synthesized hollow structures showed enhanced ORR properties (Hong et al., 2012a). Similarly, Zhang et al. (2015a) developed Pt cubic and octahedra nanocages having [100] and [111] facets, respectively, which showed higher electrocatalytic activities in terms of ORR.

However, in case of many structure sensitive reactions, introducing defects like twin boundaries may reduce the activation energy and enhance the catalytic performance (He et al., 2016). For example, Pt-enrich few atom thick nanocages showed the mass activity 7 times larger in comparison with the Pt/C catalyst as well as 4 times larger when compared with the PdPt core shell/C catalyst. However, specific activity of Pt-enrich nanocage/C was 10 times that of the Pt/C catalyst and 3.5 times that of the PdPt core shell/C catalyst. By accelerated degradation testing, the stability of Pt-enriched nanocage/C was also studied. Pt-enriched nanocage/C displayed nearly no degradation after 10,000 cycles and icosahedral Pt-enriched nanocages were stable after accelerated degradation testing.

In addition, Wang et al. (2016) benchmarked the ORR performance of Pt based icosahedral nanocages in contrast to the Pt/C catalyst. The mass as well as specific activities of Pt icosahedral nanocage showed 6.7 and 10-fold enhancement, respectively, which is ascribed to the exposure of [111] facets, twin defects, and higher distribution of Pt. However, mass activity of Pt icosahedral nanocages was enhanced 4 times after 5,000 cycles of ADT when compared with the commercial catalyst before the durability tests.

In addition to single metal NFs, fabrication of Pt based bi-and multimetallic alloy NFs could display superior activity in terms of ORR. So, alloying of Pt with transition metals like Co, Cu, Ni, etc., could induce the compressive strain on NPs' surface because of shorter Pt-Pt distance as well as downshifting of d-band center of the Pt. Additionally, downshift of the d-band center weakens the adsorption of OH species that are intermediate to ORR, facilitates the dissociation of water molecules from catalyst surface and increases the ORR reaction rate of NPs.

Dhavale and Kurungot (2015) developed a CuPt nanocage via the galvanic displacement reaction. These CuPt NCs display 2.9-fold and 2.5-fold higher levels of mass activity as well as specific activity toward oxygen reduction reaction at 0.9 V vs. reversible hydrogen electrode. In addition, stability of CuPt nanocages was investigated by a durability test under alkaline conditions. So, CuPt NCs have higher surface area and provide additional active sites for the dissociative adsorption of oxygen and improved the ORR performance. The combination of Pt and Cu play a very important role in facilitating the dissociative adsorption of O_2_. Similarly, rhombic dodecahedral Cu_3_Pt NFs synthesized by the Han group are more active toward the ORR as compared to the CuPt core shell nanostructure (Han et al., 2014).

For instance, uniform PtCu alloy hollow cubic NFs were synthesized by Wang et al., and these 3D nanoarchitectures show the larger specific as well as mass activities and improved durability toward ORR in comparison with the Pt nanocubes, commercial Pt/C as well as Pt black catalyst under alkaline solution (Wang et al., 2018a).

Lyu et al. (2017) developed the CuPt alloy RD as well as spiny CuPt RD NFs. The 3D hollow structure of both sets of NFs and abundant surface defects make them active catalysts. Toward ORR, spiny RD NFs have shown the specific activity of 1.3 and 3 times as compared to the RD NFs and Pt/C catalyst. Huang et al. (2019) have fabricated 3D RD Pt_26_Cu_74_ NFs bound with multiple index facets that were synthesized by the effective facile robust solvothermal synthetic procedure. Due to exclusive morphology and the combined effect of the two metals, these Pt_26_Cu_74_ catalysts have shown higher activity as well as stability toward ORR in comparison with the Pt_18_Cu_82_, Pt_50_Cu_50_ NC, and Pt/C catalysts.

**Figure 10:**
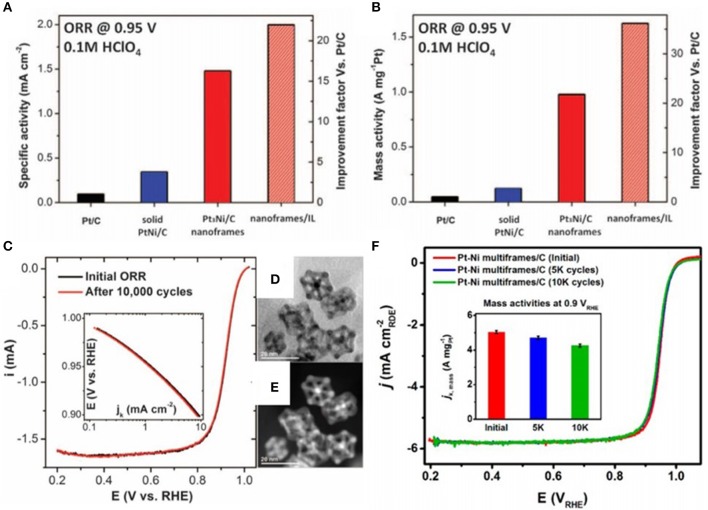
Electrochemical properties of Pt3Ni nanoframes. **(A,B)** Specific activities **(A)** and mass activities **(B)** measured at 0.95 V, and improvement factors versus Pt/C catalysts. Because of the high intrinsic activity of the Pt3Ni nanoframes, the ORR activity values are given at 0.95 V in order to avoid the extensive error margin at 0.9 V introduced by the close proximity of current values to the diffusion-limited current. IL, ionic liquid. Electrochemical durability of Pt3Ni nanoframes. **(C)** ORR polarization curves and (inset) corresponding Tafel plots of Pt3Ni frames before and after 10,000 potential cycles between 0.6 and 1.0 V. **(C)** ORR polarization curves of the Pt-Ni multiframes/C before and after 5,000 and 10,000 cycles in an O2-saturated 0.1M HClO4 solution. The inset showing changes in mass activities of the Pt-Ni multiframes/C before and after potential cycles. **(D,E)** Bright-field STEM image **(D)** and dark-field STEM image **(E)** of Pt3Ni nanoframes/C after cycles. Modified with the permission from Chen et al. (2014) Copyright 2014, American Association for the Advancement of Science. **(F)** ORR polarization curves of the Pt-Ni multiframes/C before and after 5,000 and 10,000 cycles in an O2-saturated 0.1M HClO4 solution. The inset showing changes in mass activities of the Pt-Ni multiframes/C before and after potential cycles. Modified with permission from Kwon et al. (2018a) Copyright 2018, American Chemical Society.

Controlling the structure at the atomic level can tune the catalytic performance of materials and may enhance the activity as well as durability. Han et al. developed highly active and durable Pt_3_Ni NFs as catalysts with a 3D surface and 2 nm thin platinum skin. Both internal and external surfaces of frames are available for reactants, the and presence of segregated platinum skin demonstrate the excellent ORR activity. These Pt_3_Ni NFs have exhibited enhanced mass activity and specific activity that are 36 and 22 times those of the Pt/C, respectively. Furthermore, the frame structure was retained after 10,000 cycles, even during harsh electrochemical conditions as exhibited in Figures 10A–E (Chen et al., 2014). In another report, dendrite implanted PtNi multiframes display the ECSA of 73.4 m^2^gPt^−1^ and mass activity of 1.51 A mgPt^−1^ that is the 30 time higher than the Pt/C catalyst. It is noted that the ECSA as well as ORR performance of these dendrite embedded PtNi multiframes/C are due to their porous design, several active sites, and higher index facets Figure 10F.

3D Pt anisotropy of PtNi RD was tuned by controlling the ratio of Pt and Ni precursors—as a result, entire hollow NFs and excavated NFs were gained. These excavated NFs display the ~10 times greater specific as well as ~6 times larger mass activity in terms of ORR as compared to the Pt/C and double the mass activity of hollow NFs. However, the higher activity is due to more Ni content in the near-surface region and 2D sheet structure within the NFs, which minimized the number of buried Pt sites (Becknell et al., 2017). Wang et al. explained the preferential removal of Ni components of the tetrahexahedral (THH) PtNi NPs by using the CO. These PtNi (THH) NF structures demonstrated 8-fold higher ORR mass activity than in commercial Pt/C. In another report, PtNi NFs along (THH) and RD morphologies were used for the ORR. The as synthesized PtNi (THH) NFs display more enhanced ORR activity that shows 20.9 times larger mass activity in comparison with the commercial Pt/C.

It is observed that in addition to structure, composition also plays a very important role for the enhanced catalytic performance. For instance, RD PtCuNi NF catalysts display 6-fold higher mass activity as compared to commercial Pt/C in terms of ORR. Hollow PtPdRu dendrite nanocages with porous structure not only showed 3D surface active sites from both interior and exterior surfaces but also improved the resistance to particle aggregation. In addition, mass transfer of species inside the porous nanocages also increased. However, electronic as well as strain effect of three metal components also enhanced the activity. Therefore, trimetallic dendritic nanocages accelerate the kinetics of oxygen reduction. In comparison with the Pt/C, ECSA, mass activity as well as specific activity of PtPdRu dendritic nanocage were higher (Huang et al., 2018).

The PtPdNi mesoporous truncated octahedral nanocage showed higher catalytic performance toward the ORR relative to the core shell mesoporous truncated octahedra and Pt/C catalyst due to their mesoporous surface, hollow design, polyhedral shape (Wang et al., 2019).

### Bifunctional Catalysts

As mentioned above, the Pt based alloy nanoarchitectures show excellent catalytic performance toward ORR and MOR. However, the bifunctional catalysts show more importance because they function as active catalysts in both anodes as well as cathodes of DMFCs. Among different hollow structures, NFs have received more attention because of their open 3D nanoframe structure. For instance, Zhang et al. (2016) synthesized the highly anisotropic 5-fold twin CuPt NFs, which shows more ORR as well as MOR activity, with a specific activity of 1.71 mA ${\text{cm}}_{\text{Pt}}^{-2}$ toward ORR and 18.2 mA ${\text{cm}}_{\text{Pt}}^{-2}$ for MOR. However, the rich concave site along with a higher coordination number demonstrated superior ORR activity. The most important point in this research is that the nanothorns on the edges as well as higher index facets provided extra active surface area.

**Figure 11:**
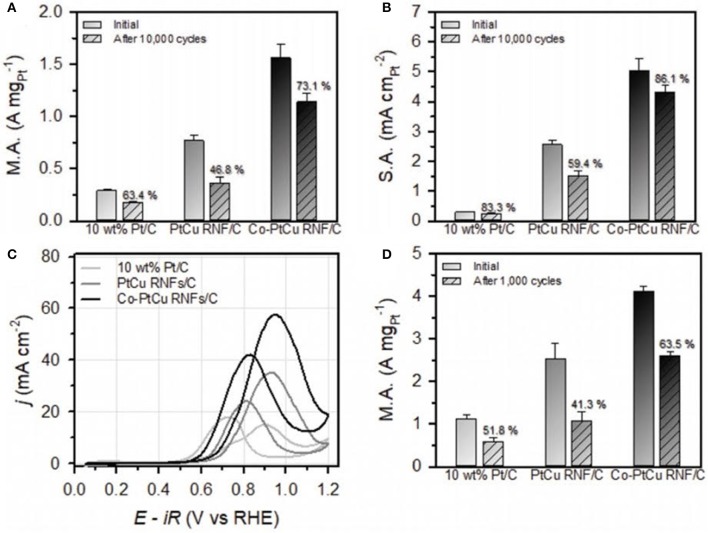
**(A,B)** ORR mass and specific activities of electrocatalysts, before and after 10,000 ORR ADT cycles. **(C)** CVs of Co-PtCu RNF/C, PtCu RNF/C, and Pt/C catalysts in 0.1 M HClO_4_+1 M CH_3_ OH solution. **(D)** Mass activity of electrocatalysts for the MOR, before and after the ADTs. Modified with permission from Kwon et al. (2018b) Copyright 2018, WILEY-VCH Verlag GmbH & Co. KGaA, Weinheim.

In another report, Kwon et al. (2018b) fabricated Co-doped CuPt RD NFs which displayed a higher MOR specific activity of 13.3 mA ${\text{cm}}_{\text{Pt}}^{-2}$ as well as a mass activity of 4.11 A ${\text{mg}}_{\text{Pt}}^{-1}$. In addition, ORR performance was also boosted with a specific activity of 5.03 mA ${\text{cm}}_{\text{Pt}}^{-2}$ as well as a mass activity of 1.56 A ${\text{mg}}_{\text{Pt}}^{-1}$. However, protruded vertices also play a most important role in boosting the durability, which is confirmed by the study with the un-doped CuPt NFs (Figure 11). Another example is the highly composition-segregated PtNi NFs with open frame structure transformed from solid PtNi NCs. According to the catalytic results it is proven that these PtNi NFs have shown excellent catalytic performance toward ORR as well as alcohol oxidation in comparison with the commercial Pt/C catalyst. Other Pt based nanoarchitectures have been studied which are active as bifunctional catalysts in terms of ORR at the cathode as well as other fuel oxidation reactions at the anode like formic acid, ethanol, glycerol and ethylene glycol (Ye et al., 2017).

Pt_3_Ni tetrahexaherdal NFs were developed by CO etching which is highly open, stable as and has a higher index microstructure which consists of a segregated Pt thin surface and downshifted d-band center, which is revealed by the Density Functional Theory (DFT). These NFs showed excellent catalytic performance, such as higher stability in harsh reaction conditions, which promoted direct electrooxidation of formic acid to CO_2_ and enhanced ORR activities. FAOR occurs on the Pt based electrode and proceeds via a dual path mechanism. In the first pathway, direct oxidation generates CO_2_ and in the second pathway there is a generation of CO adsorption and then oxidation of CO_ads_ to CO_2_ at higher potentials. Very recently, Wang et al. used Co etching at higher temperatures for the fabrication of Pt_3_Ni tetrahexahedron NFs by using the facile as well as efficient method. So, these NFs revealed excellent stability under prolonged electrochemical potential cycles (Wang et al., 2017a).

## Conclusion and Perspectives

In recent years, noble metal-based NFs have received more attention because of their special structure as well as their physicochemical properties. For controlled synthesis and advanced applications of noble metal-based NFs, great efforts have been devoted in recent years. According to this review, we explained distinct methods such as the photocatalytic template method, electrodeposition, the Kirkendall effect, galvanic replacement, oxidative/chemical etching, and other methods for the fabrication of noble metal-based alloy NFs. In addition, different shapes of noble metal-based NFs have been developed and focused with ultrathin walls, high index facets, twin boundaries compositional segregation, and inner structural support. These noble metal-based NFs show excellent performance in fuel cells.

However, for the fabrication of noble metal-based NFs still more challenges are present such as tuning the porosity, voids, elemental composition as well as the thickness of ridges and atomic arrangement. For catalytic properties, controlling the ridge thickness with segregated Pt skin of metal-based NFs is of great importance and challenging. To preserve the stability of NFs after electrocatalytic cycling is a difficult task. To improve the stability of such nanostructures, the addition of dopant, or another stable structure inside NFs may secure the atomic arrangements and stability as well. The addition of a doping material can enhance the exposed atoms on the surface. NFs with more than three or four metals and other doping materials can further increase the activities and stabilities.

Furthermore, the implementation of theoretical studies must be considered to gain an understanding of the actual basis of the catalytic properties of multimetallic NFs. Additionally, theoretical studies should focus on the calculation of surface energies of the exposed atoms. It should also calculate the binding energies of reaction intermediates and surfaces of the electrocatalyst for multi-metallic structures. Density Functional Theory studies have been used for the few faceted bimetallic structures, but for the multimetallic NFs these studies are very rare. There is an urgent need to find easy calculation strategies for understanding multimetllic NFs with complex atomic arrangements, which can help to clearly identify the reasons behind their enhancement of activity and stability. Another necessity is to understand the rearrangements of atoms at atomic scale during the process of electrocatalysis. For this purpose, *in situ* study of electrochemical reactions may be a solution for the deep understanding of phase segregation and arrangement of atoms. Additionally, these catalysts should be reproducible at industrial level and should withstand the real operating environment.

Sometimes, the thermodynamic stability of alloy phases varies during synthesis and electrochemical cycling. Few efforts have been made to perform elecrocatalysis at higher potentials and temperatures to check the stability of catalysts under real operating conditions. In addition, hollow structures show a different electrochemical active surface area at different over potentials due to mass transportation and make its use limited in real applications. At low over potential, O_2_ diffuses inside the inner part of hollow structures, and at high over potential, O_2_ diffuses slowly inside the voids. Some groups proposed that the use of ionic liquids in place of aqueous liquids could fill the interior void of NFs and offer more O_2_ solubility that could be beneficial for a real operating environment for fuel cells.

## Author Contributions

FN has substantial contributions to the conception, drafting and writing of this work. AS helped in writing the draft. TA contributed to the writing and revising it critically for important intellectual content. NH reviewed it and contributed in its revision. All authors read and agreed the final version of the manuscript.

### Conflict of Interest Statement

The authors declare that the research was conducted in the absence of any commercial or financial relationships that could be construed as a potential conflict of interest.
